# Multi-omics insights into tumor grade progression in clear cell renal cell carcinoma: from molecular mechanisms to precision therapeutics

**DOI:** 10.3389/fcell.2026.1815377

**Published:** 2026-04-20

**Authors:** Rajat Subhra Jena, Akhilesh Mishra

**Affiliations:** Computational Oncology Laboratory, Department of Life Sciences, National Institute of Technology Rourkela, Sundargarh, Odisha, India

**Keywords:** clear cell renal cell carcinoma, immune exhaustion, metabolic reprogramming, multi-omics integration, precision oncology, spatial tumor microenvironment, tumor grade progression

## Abstract

Clear cell renal cell carcinoma (ccRCC) can transition from indolent, low-grade lesions to high-grade, lethal disease through a layered cascade of genomic, epigenomic, metabolic, and immune remodeling. The initiating event in ∼90% of ccRCC is loss of chromosome 3p, enabling biallelic inactivation of VHL and frequent co-loss of chromatin regulators PBRM1, BAP1, and SETD2. The order and combination of genetic alterations shape distinct evolutionary trajectories in ccRCC. PBRM1 loss, observed in approximately 55% of cases, is linked to angiogenic, initially low-grade tumors that may later progress to higher-grade disease. In contrast, BAP1 loss (∼15%) drives early high-grade, inflammatory, immune-enriched phenotypes associated with aggressive behavior and worse prognosis. Progression is further shaped by structural and copy-number events including, chromothripsis coupling 3p loss with 5q gain, and recurrent 9p and 14q losses and 8q gain further promote cell-cycle dysregulation, genomic instability, and metastatic competence. Functionally, VHL loss stabilizes HIF-2α, driving VEGF signaling and Carbonic Anhydrase IX (CA9) expression and coupling pseudohypoxia to metabolic reprogramming and redox protection (glutathione/SLC7A11). Proteogenomic and metabolomic studies further highlight nutrient addiction with GLUT1/ASCT2 upregulation and a stress-resistant metabolic shield linked to grade and therapy resistance. Single-cell and spatial atlases place these programs in anatomic setting. They show that invasive fronts with high epithelial–mesenchymal transition (EMT) activity co-localize with myeloid and regulatory T-cell niches dominated by IL-1β, NF-κB, IL-10, STAT3, and TGF-β, along with exhausted CD8^+^ T cells, thereby promoting immune escape and invasion. Integrating these layers yields mechanism-based biomarkers and therapeutic nodes for risk-adapted precision treatment.

## 1 Introduction

Tumor grade is among the strongest prognostic variables in ccRCC, yet grade is a phenotype rather than a single lesion. It reflects how tumor cells and their microenvironment co-evolve to sustain proliferation, invasion and immune escape. Clinically, grade helps guide surveillance and adjuvant strategies, but it is an imperfect proxy for the biology that ultimately drives recurrence and metastasis. After nephrectomy, 5-year cumulative recurrence rates have been reported to range from ∼7% to >60% depending on clinicopathological risk category, emphasizing the need for biomarkers that anticipate aggressive evolution rather than only describe it after the fact (Marchioni et al., 2021).

Multi-omics technologies now allow grade progression to be reframed as an ecosystem problem: truncal genomic events set a permissive landscape; epigenomic and non-coding programs rewire transcriptional outputs; metabolic remodeling supplies energy and redox control; and the tumor-immune microenvironment (TIME) selects for immune-evasive, invasive clones (Hsieh et al., 2017; Gavi et al., 2025). The translational challenge is integration, specifically in identifying robust, clinically measurable features that reflect these layered processes and reveal actionable targets.

**FIGURE 1:**
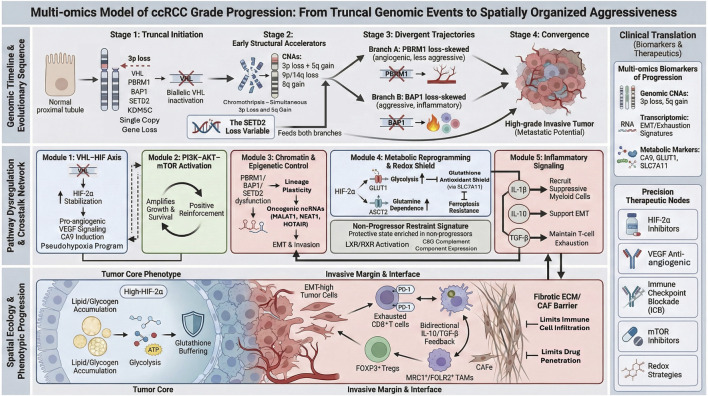
Integrated multi-omics model of ccRCC grade progression. The top panel depicts early truncal genomic events including 3p loss with VHL and chromatin-regulator inactivation followed by CNA/structural evolution through chromothripsis (simultaneous 3p loss and 5q gain), 9p/14q loss, 8q gain, and divergent PBRM1-angiogenic *versus* BAP1/SETD2-aggressive trajectories. The middle panel summarizes pathway crosstalk: VHL loss stabilizes HIF-2α to drive VEGF/CA9, metabolic rewiring and redox buffering (SLC7A11–glutathione), reinforced by PI3K–AKT–mTOR signaling and inflammatory circuits (IL-1β/NF-κB, IL-10/STAT3, TGF-β). The bottom panel maps spatial phenotypes, with metabolic cores and EMT/immune-suppressive invasive margins enriched for TAMs/Tregs and exhausted CD8^+^ T cells. Biomarker and therapeutic nodes are aligned to each module.

In parallel, non-invasive approaches such as radiomics are beginning to approximate grade preoperatively; MRI-based radiomics models can differentiate high-grade from low-grade ccRCC with pooled AUC ∼0.84, and combined radiomics-clinical models approach AUC ∼0.90 in meta-analysis (Broomand Lomer et al., 2025). The opportunity now is to align imaging, tissue and liquid biomarkers with mechanistic multi-omics to enable risk-adapted surveillance and precision therapeutics (Figure 1).

## 2 Multi-omics model of tumor grade progression in ccRCC

### 2.1 Genomic foundations: truncal 3p loss, branching evolution and copy-number acceleration

A consistent genomic theme in ccRCC is early chromosomal disruption. TCGA analyses established that loss of chromosome 3p is near-universal and seeds inactivation of core tumor suppressors on that arm, including VHL, PBRM1, BAP1, and SETD2 (Creighton et al., 2013). Hsieh and colleagues conceptualized 3p loss as the initiating scaffold that links VHL-driven hypoxia signaling, chromatin dysregulation and downstream phenotypes central to ccRCC biology, providing a unifying framework for how early events can echo through later grade progression (Hsieh et al., 2018). These early lesions are typically truncal (shared by all regions), but they do not dictate a single path. Instead, they create biased evolutionary trajectories.

In a seminal analysis, BAP1-mutation was reported in ∼15% of ccRCC tumors and is associated with higher grade tumor having aggressive behavior and worse prognosis, whereas PBRM1-mutation was reported in ∼55% of tumors and is enriched in less aggressive phenotypes, that may later progress to higher grade tumors (Kapur et al., 2013; Gerlinger et al., 2012). Notably, BAP1 and PBRM1 mutations are largely mutually exclusive, consistent with distinct evolutionary trajectories. Although concurrent BAP1/PBRM1 dual loss is rare and leads to the most aggressive phenotypes and the poorest survival outcomes, available evidence suggests that these tumors more likely represent late evolutionary convergence or sub-clonal acquisition of a second chromatin-regulator defect, rather than a common founding genotype (Turajlic et al., 2018; Xie et al., 2025). Conceptually, these alterations define parallel evolutionary trajectories in ccRCC tumorigenesis. Tumors harboring PBRM1 mutations often follow a relatively indolent, angiogenesis-dominant course that may progress in grade through the acquisition of additional drivers, whereas BAP1-mutant tumors frequently exhibit aggressive transcriptional programs and higher-grade histology early in tumor evolution (Kapur et al., 2013; Turajlic et al., 2018). These genotype-defined groups also carry important therapeutic implications, as tumors with PBRM1 alterations are often associated with angiogenic signaling pathways responding to VEGF-targeted therapies, whereas BAP1-mutant tumors frequently display immune-inflamed transcriptional states that may respond to immunotherapy-based regimens (Kapur et al., 2013; Jonasch et al., 2021a).

Multi-region sequencing revealed that ccRCC is highly heterogeneous and evolves through branched phylogenies, allowing high-grade subclones to emerge within an otherwise lower-grade tumor (Gerlinger et al., 2012). Phylogenetic timing suggests that truncal initiating events can precede diagnosis by years to decades, creating a long window for subclonal diversification and, in some tumors, punctuated copy-number gains and losses that accelerate malignant progression (Turajlic et al., 2018). Whole-genome sequencing has also highlighted catastrophic rearrangements (chromothripsis) that can couple early 3p loss with 5q gain, reinforcing the view that ccRCC can undergo a long period of evolutionary development before becoming clinically detectable (Turajlic et al., 2018; Jonasch et al., 2021a).

Copy-number alterations provide a quantitative bridge between genotype and grade. Losses on 9p and 14q, and gains on 8q, are repeatedly linked to aggressive behavior and poor outcome, and they often appear as later, subclonal events that accompany progression toward high-grade disease (Creighton et al., 2013; Turajlic et al., 2018; Jonasch et al., 2021a). A practical controversy is whether high-grade disease is “pre-wired” early (for example, by BAP1 loss) or is largely a late product of copy-number acceleration and microenvironmental selection. Increasingly, the field is converging on a hybrid view: truncal drivers define a baseline state, while later aneuploidy and the TIME determine which clones dominate (Jonasch et al., 2021a).

High-grade-focused biomarker studies illustrate how genomic states translate into measurable molecular targets. In high-grade ccRCC, overexpression of BCL9 and TPX2 has been associated with adverse outcomes; high BCL9 correlated with poorer progression-free survival (HR ∼2.9) and high TPX2 with worse overall survival (HR ∼5.5) (Kasperczak et al., 2025a).

Similarly, prominin-1 (PROM1) and the splicing factor elongation factor Tu GTP-binding domain-containing protein 2 (EFTUD2) show heterogeneous expression within high-grade tumors; low EFTUD2 immunohistochemistry scores associated with worse progression-free survival (HR ∼5.0) (Kasperczak et al., 2025b). Together, these studies exemplify a growing trend: shifting from single-driver narratives to multi-layer biomarker panels anchored in functional states (proliferation, splicing, invasion).

### 2.2 Epigenomic and non-coding RNA programs

Genetic lesions alone incompletely explain ccRCC grade progression because many key drivers (PBRM1, BAP1, SETD2) are chromatin regulators and their impact is executed through altered transcriptional and epigenetic states (Hsieh et al., 2018). Non-coding RNAs (ncRNAs) add an additional regulatory layer that can be both mechanistic and clinically measurable.

Large-scale profiling has identified numerous long non-coding RNAs (lncRNAs) and microRNAs that associate with pathological stage, grade and outcome, although few have reached clinical-grade validation (Tesarova et al., 2025). Examples repeatedly implicated across datasets include metastasis associated lung adenocarcinoma transcript 1 (MALAT1), nuclear enriched abundant transcript 1 (NEAT1) and HOX transcript antisense RNA (HOTAIR), which can act through chromatin regulation, RNA processing, and gene-network modulation (Tesarova et al., 2025; Liu et al., 2026).

Mechanistically, lncRNAs can scaffold chromatin-modifying complexes, modulate enhancer activity, or act as competing endogenous RNAs; collectively, these functions reshape programs controlling differentiation, motility, and therapy resistance (Tesarova et al., 2025; Liu et al., 2026). EMT is a central convergence point: it is repeatedly enriched in high-grade and invasive regions, and ncRNA networks regulate EMT through canonical pathways (for example, PI3K-AKT, mitogen-activated protein kinase (MAPK), wingless-related integration site (WNT)/β-catenin and TGF-β signaling) and direct modulation of epithelial and mesenchymal transcription factors (Liu et al., 2026). A key debate is whether EMT in ccRCC represents a binary switch or a spectrum of partial EMT states; spatial and single-cell data increasingly support the latter, with hybrid epithelial-mesenchymal phenotypes at invasive fronts.

Human microRNA-21-5p (hsa-miR-21-5p) is upregulated in ccRCC, associates with poor overall survival and, in a model validated using grade and stage, correlates with immune-evasion features and reduced predicted immunotherapy responses (Xu et al., 2025). Higher miR-21-5p expression was also linked to differential drug sensitivity profiles, including vascular endothelial growth factor receptor–tyrosine kinase inhibitors (VEGFR-TKIs), raising the possibility that ncRNA readouts could inform therapy selection in defined contexts (Xu et al., 2025). The current gap is clinical standardization: ncRNA assays are sensitive to pre-analytical variables and tumor sampling, and it remains unclear which signatures are robust across platforms, sites and treatment settings.

### 2.3 Metabolic and proteomic reprogramming

ccRCC is often described as a metabolic malignancy because VHL loss stabilizes hypoxia-inducible factors and reshapes carbon and lipid metabolism. Multi-omic profiling has linked disease progression to coordinated metabolic reprogramming, including increased glycolysis and pentose phosphate pathway activity, altered mitochondrial programs, and enhanced nutrient acquisition (Hu et al., 2024; Clark et al., 2019). These shifts are not cosmetic; they support proliferation, biosynthesis and oxidative stress tolerance - traits that become increasingly important as tumors progress to higher grade and face immune and therapeutic pressures.

Across multi-omic datasets, aggressive ccRCC frequently shows increased expression of nutrient transporters that enable high-flux metabolism, including GLUT1 (SLC2A1) for glucose and ASCT2 (SLC1A5) for glutamine, consistent with the metabolic demands of high-grade tumors (Hu et al., 2024; Clark et al., 2019).

Grade-stratified metabolomics adds granularity. In tumor samples spanning grades G2 to G4 (n = 14, 12 and 9, respectively), ccRCC showed accumulation of sphingosine, sphingosine-1-phosphate (S1P) and ceramides relative to adjacent kidney. Notably, dihydrosphingosine and dihydroceramide accumulation was largely restricted to higher grades, implicating sphingolipid flux as a grade-associated metabolic layer (Mlynarczyk et al., 2022). These findings complement proteogenomic evidence that aggressive tumors adopt stress-resistant states, frequently characterized by strengthened redox buffering and upregulation of transporters that supply glucose and glutamine (Hu et al., 2024; Clark et al., 2019).

Spatial context matters for metabolism. Integrated spatial analyses suggest that metabolic programs can be anatomically partitioned, with tumor epicenters showing heightened core metabolic activity and peripheral regions enriched for vasculogenesis, inflammatory cues and EMT programs (Gavi et al., 2025). This observation motivates a clinical caution: bulk metabolomics or proteomics can average away the invasive niche most relevant to grade escalation and metastasis.

New mechanistic targets are emerging at the interface of metabolism and cell death. Kringle containing transmembrane protein 2 (KRM2) is upregulated in RCC and promotes proliferation and migration while inhibiting ferroptosis through interaction with activating transcription factor 2 (ATF2); ATF2 silencing reverses the cancer-promoting and ferroptosis-inhibiting effects of KRM2 (Liu et al., 2025). Ferroptosis vulnerability is attractive for high-grade ccRCC because redox-adapted tumors may rely on glutathione and lipid peroxidation control. However, whether ferroptosis is broadly targetable in ccRCC remains unresolved and will require biomarker-driven trials.

### 2.4 Tumor-immune ecosystem and spatial organization

ccRCC is paradoxical: it is often heavily infiltrated by immune cells yet can remain profoundly immunosuppressed. Single-cell and imaging-based atlases have delineated complex immune architectures, including exhausted CD8^+^ T cells, forkhead box P3 (FOXP3+) regulatory T cells (Tregs), and diverse tumor-associated macrophage states (Chevrier et al., 2017).

Spatial and single-cell transcriptomics add a key refinement: immunosuppressive interactions concentrate at specific anatomical sites, especially the tumor-normal interface where invasion occurs (Gavi et al., 2025; Song et al., 2025). Song and colleagues identified an IL-1beta/IL-18-expressing Treg subset that interacts with mannose receptor C-type 1 (MRC1+)/folate receptor beta (FOLR2+) tumor-associated macrophages at the tumor-normal interface, reinforcing an immunosuppressive milieu linked to tumor progression (Song et al., 2025). In aggressive tumors, cancer-associated fibroblasts (CAFs) remodel extracellular matrix (ECM) into a fibrotic barrier that blocks immune infiltration and drug penetration, constructing an immunosuppressive niche that sustains progression. Functionally, these niches coincide with T-cell exhaustion programs including programmed cell death protein-1 (PD-1), lymphocyte activation gene-3 (LAG-3), and T-cell immunoglobulin and mucin domain-3 (TIM-3)–associated transcriptional modules, and with EMT at the invasive edge, providing a spatial explanation for why immune infiltration does not necessarily translate into immune control (Chevrier et al., 2017; Song et al., 2025).

Beyond T cells and macrophages, the role of B cells in ccRCC has become a rapidly evolving. While tertiary lymphoid structures (TLS) and B-cell-rich niches can mark productive anti-tumor immunity in some contexts, regulatory B cells (Bregs) can instead support immune suppression. Importantly, the prognostic effect of B-cell-rich niches likely depends on TLS organization and maturity, as mature germinal center-positive TLS are generally associated with effective antitumor immunity, whereas immature or poorly organized aggregates may be less protective and can coexist with suppressive B-cell programs (Fridman et al., 2022; Fridman et al., 2023). Proteomic profiling of non-progressing (NP) tumors shows a protective signature, with proteins like C8G being abundant and pathways like liver X receptor (LXR)/retinoid X receptor (RXR) activation and the Acute Phase Response being active, suggesting successful immune surveillance. In ccRCC, elevated Breg signatures correlated with higher grade and worse progression-free survival. Spatial analyses localized CD20^+^CD23+IL-10+ Bregs within immunosuppressive ccRCC microenvironment (with or without tertiary lymphoid structures), while CMLBregS signature identified high-risk, immunotherapy-refractory tumors with poorer responses to immune checkpoint blockade (Ge et al., 2025).

Therapeutically relevant immune-tumor crosstalk can also be driven by receptor tyrosine kinases linked to aggressive disease. In a cohort of 110 primary ccRCC, AXL receptor tyrosine kinase (AXL) expression correlated with primary tumor stage, while cellular mesenchymal–epithelial transition factor (c-MET) expression correlated with distant metastasis, histological grade and overall survival. High c-MET expression was associated with increased programmed death-ligand 1 (PD-L1)+ tumor-infiltrating immune cells, and both AXL and c-MET were upregulated in sunitinib-treated tissues, suggesting a role in VEGFR-TKI resistance and immune modulation (Mikami et al., 2025). This provides mechanistic context for the clinical activity of cabozantinib, which targets VEGFR as well as AXL and MET.

## 3 Biomarker layers informing precision therapeutics

Multi-omics integration is essential to understand how ccRCC progresses from low-to high-grade disease. Genomic studies define early truncal events, notably 3p loss with VHL inactivation and recurrent PBRM1, BAP1, and SETD2 alterations, which establish distinct evolutionary routes. Bulk and single-cell transcriptomics translate these lesions into angiogenic, immune-inflamed, proliferative, and EMT-associated cell states. Proteogenomic and metabolomic profiling then identify the functional consequences of these states, including HIF-driven glycolysis, pentose phosphate pathway activation, glutamine dependence, lipid remodeling, and glutathione-based redox protection. Spatial transcriptomics and multiplex imaging localize these programs within tissue architecture, revealing metabolically active cores and invasive, immunosuppressive margins enriched for exhausted CD8^+^ T cells, regulatory T cells, macrophages, and fibroblasts. Together, these integrated layers generate clinically actionable biomarkers and therapeutic vulnerabilities for precision oncology (Hsieh et al., 2017; Gavi et al., 2025; Clark et al., 2019). Multi-omics integration becomes even more clinically meaningful when it yields practical biomarkers and testable therapeutic hypotheses. Current evidence supports several complementary biomarker layers for grade-progression risk stratification:

- Truncal genomic risk: 3p loss with copy-number alterations including 9p and 14q loss, 8q gain that mark evolutionary acceleration (Creighton et al., 2013; Hsieh et al., 2018; Kapur et al., 2013; Gerlinger et al., 2012; Turajlic et al., 2018; Xie et al., 2025; Jonasch et al., 2021a).
- Regulatory state biomarkers: EMT-associated transcriptional programs and ncRNA signatures (for example, miR-21-5p) that report on invasion potential and immune evasion (Liu et al., 2026; Xu et al., 2025).
- Metabolic state biomarkers: Expression of HIF-responsive markers such as Carbonic Anhydrase IX (CAIX/CA9), nutrient transporters, and lipid/redox pathways (including sphingolipid alterations and ferroptosis regulators) that reflect stress resistance and may inform metabolic targeting (Hu et al., 2024; Clark et al., 2019; Mlynarczyk et al., 2022; Liu et al., 2025; Wong et al., 2025).
- Immune ecosystem biomarkers: Spatially resolved exhaustion and immunosuppression signatures (Tregs, exhausted CD8^+^ T cells, MRC1+FOLR2+ macrophages, Bregs) that predict immune checkpoint sensitivity and identify combination strategies (Chevrier et al., 2017; Song et al., 2025; Fridman et al., 2022; Fridman et al., 2023; Ge et al., 2025; Chen et al., 2026).

How might these layers guide therapy? Three pillars dominate ccRCC treatment and intersect with grade biology. First, HIF-2α blockade targets the metabolic consequences of VHL loss; belzutifan has demonstrated clinical activity in von Hippel-Lindau disease-associated RCC and illustrates the tractability of the HIF axis (Jonasch et al., 2021b). Second, VEGF/angiogenesis inhibition remains foundational, particularly in angiogenic transcriptional states enriched for PBRM1-mutant tumors, and multi-target TKIs (for example, cabozantinib) may be especially relevant when AXL/MET programs or VEGFR-TKI resistance features are present (Mikami et al., 2025; Chen et al., 2026). Third, immune checkpoint blockade is central for inflamed phenotypes but is blunted by exhaustion and immunosuppressive niches; emerging strategies include next-generation checkpoint targets such as lymphocyte activation gene-3 (LAG-3), T-cell immunoglobulin and mucin domain-3 (TIM-3), and T-cell immunoreceptor with Ig and ITIM domains (TIGIT), as well as bispecific antibodies and cytokine-based approaches (Chen et al., 2026).

Later-line therapy choices can also be contextualized by multi-omic state, in a systematic review of post-immune checkpoint inhibitor (ICI) metastatic RCC, lenvatinib plus everolimus produced median progression-free survival typically ∼6–7 months and objective response rates spanning ∼14–56%, albeit in heterogeneous, mostly non-randomized datasets, highlighting the need for predictive biomarkers for sequencing decisions (Iovane et al., 2026).

A recurrent translational pitfall is sampling bias. RNA and protein signatures can vary substantially by tumor region and sample site, which can confound biomarker development if spatial context is ignored (Gavi et al., 2025). Practical solutions are emerging: radiogenomics to guide sampling; multiplex immunohistochemistry to quantify immune niches; and liquid biopsy for longitudinal monitoring. Yet prospective validation and clinical decision thresholds remain rare (Table 1).

**TABLE 1:** Multi-omics biomarkers and therapeutic opportunities for ccRCC grade progression.

Omics layer	Representative biomarkers/features	Link to grade progression	Translational implication
Genomics/copy number alteration (CNAs)	3p loss with VHL plus BAP1 or PBRM1; later 9p/14q loss; 8q gain (Creighton et al., 2013; Hsieh et al., 2018; Kapur et al., 2013; Gerlinger et al., 2012; Turajlic et al., 2018; Xie et al., 2025)	Truncal driver context plus copy-number acceleration associates with higher grade, metastasis risk and poor outcome	Risk stratification; trial enrichment; informs expectation of angiogenic vs aggressive trajectories
Proteomics/signaling	BCL9, TPX2; EFTUD2; AXL and c-MET (Kasperczak et al., 2025a; Kasperczak et al., 2025b; Mikami et al., 2025)	Overexpression linked to adverse survival or progression; AXL/MET linked to high grade and VEGFR-TKI resistance	Candidate biomarkers; supports MET/AXL-targeted strategies (e.g., cabozantinib) and exploration of RNA-processing vulnerabilities
ncRNA/gene regulation	lncRNA and microRNA panels; miR-21-5p (Tesarova et al., 2025; Liu et al., 2026; Xu et al., 2025)	Associations with grade, stage, EMT programs and immune evasion; limited clinical standardization	Potential prognostic adjunct; future liquid biopsy targets; may help therapy selection once validated
Metabolism	Glycolysis/PPP shift; sphingolipid remodeling (S1P, ceramides; higher-grade dihydroceramides); ferroptosis regulators (KRM2-ATF2) (Hu et al., 2024; Clark et al., 2019; Mlynarczyk et al., 2022; Liu et al., 2025)	Stress tolerance and redox adaptation increase with progression; grade-dependent lipid flux changes	Supports HIF-axis targeting (HIF-2alpha inhibitors), metabolic targeting and exploration of ferroptosis-inducing combinations
Immune and spatial ecology	Exhausted CD8^+^ T cells; IL-1beta/IL-18 Tregs with MRC1+FOLR2+ macrophages; Breg signature (CMLBregS) (Chevrier et al., 2017; Ge et al., 2025)	Immunosuppressive niches concentrate at invasive fronts and associate with higher grade and reduced immune checkpoint inhibitor (ICI) benefit	Rational ICI combinations (next-generation checkpoints, myeloid/Treg/Breg targeting); spatial biomarkers to guide selection
Imaging (radiomics)	MRI radiomics models for high-grade vs low-grade discrimination (Broomand Lomer et al., 2025)	Noninvasive approximation of grade; complements tissue sampling	Preoperative risk assessment; sampling guidance; surveillance tailoring

## 4 Discussion, controversies, gaps and next steps

Several conceptual tensions shape the field. One concerns determinism *versus* plasticity: to what extent is high-grade biology encoded by early truncal drivers (for example, BAP1 loss) *versus* produced by later copy-number upheaval and therapy-educated microenvironments? Another concerns immune infiltration: why can abundant CD8^+^ T cells coexist with poor outcomes: a paradox increasingly resolved by spatially localized exhaustion and suppressive niches but still challenging for bulk biomarker assays. A third concerns the role of B cells and tertiary lymphoid structures, where opposing observations (beneficial immune organization vs regulatory immune suppression) likely reflect cell-state heterogeneity rather than true disagreement.

Key research gaps are clear. First, true longitudinal multi-omics across grade progression in the same patient is scarce; most datasets are cross-sectional and confounded by inter-patient heterogeneity. Second, spatial multi-omics is still difficult to scale, and single-cell metabolomics remains early, limiting our ability to connect metabolic phenotypes to local immune interactions. Third, most candidate biomarkers lack prospective validation, assay harmonization and predefined decision thresholds.

Looking ahead, three developments could shift the field. (i) Risk-adapted clinical trials that embed multi-omic profiling (including spatial readouts) to test mechanism-matched combinations: for example, pairing ICIs with myeloid/Treg-targeting or metabolic/ferroptosis strategies in immune-suppressed, redox-adapted high-grade tumors. (ii) Integrated diagnostic pipelines that couple imaging (radiomics and molecular PET), digital pathology and targeted multi-omic panels to deliver interpretable, clinic-ready grade progression reports. (iii) Patient-centered monitoring using liquid biopsy and immune/metabolic biomarkers to detect evolutionary acceleration before radiographic progression, enabling earlier intervention.

In summary, ccRCC grade progression reflects a coordinated transition in genomic architecture, gene regulation, metabolism and spatially organized immune ecology. Multi-omics integration can convert this complexity into actionable biomarkers and rational combination therapies, moving beyond one-size-fits-all treatment toward risk-adapted, mechanism-guided precision oncology.
